# Machine Learning Application to Predict Bicycle Ergometer Test Results: a Prospective Cohort Study

**DOI:** 10.17691/stm2026.18.2.06

**Published:** 2026-04-30

**Authors:** E.V. Berezina, K.A. Blinova, O.A. Dmitrieva, I.E. Mishina

**Affiliations:** DSc, Head of the Department of Physics, Chemistry, and Mathematics; Ivanovo State Medical University, 8 Sheremetevsky Prospect, Ivanovo, 153012, Russia; MD, PhD, Associate Professor, Department of Oncology and Radiation Therapy; Ivanovo State Medical University, 8 Sheremetevsky Prospect, Ivanovo, 153012, Russia; PhD, Researcher; G.A. Krestov Institute of Solution Chemistry of the Russian Academy of Sciences, 1 Akademicheskaya St., Ivanovo, 153045, Russia; MD, DSc, Professor, Department of Postgraduate Medical Education; First Deputy Director of Medical Institute; Saint Petersburg State University, 7/9 Universitetskaya naberezhnaya, Saint Petersburg, 199034, Russia

**Keywords:** machine learning, bicycle ergometry test, six-minute walk test, cardiac rehabilitation, prediction, gradient boosting

## Abstract

**The aim of the study** was to develop an optimal technique to predict the results of a bicycle ergometry test (BET) based on the parameters recorded during a six-minute walk test (6MWT) using machine learning methods.

**Materials and Methods:**

The study involved 56 patients who had experienced acute myocardial infarction and were undergoing the second stage of cardiac rehabilitation. The patients underwent a complete examination, including history taking, physical examination, anthropometric assessment, as well as a symptom-limited BET and 6MWT. During the 6MWT, we recorded the following: the distance covered, the heart rate, the blood pressure, the oxygen saturation, Borg rating of perceived exertion, the number of steps taken, and the electrocardiographic data. The algorithms for random forest, gradient boosting, k-nearest neighbors, and multiple linear regression were used to construct the machine learning models. The performance of the models was evaluated based on a determination coefficient, a mean absolute error, a mean square error, and a root mean square error. SHAP analysis was applied to interpret the findings.

**Results:**

The gradient boosting model provided the best prediction quality with a high determination coefficient (R^2^ being around 0.99) and low error values for both target metrics: the distance walked in the 6MWT and the metabolic equivalent achieved during the BET. The significance analysis of features revealed the heart rate, age, and the body mass index to have the greatest impact on predicting the 6MWT distance, while for predicting the metabolic equivalent, the distance covered, the number of steps, and the body mass index were the most significant.

**Conclusion:**

The developed gradient boosting-based machine learning model demonstrated its high efficiency in predicting the results of the 6MWT-based BET. The suggested method can serve as a valuable auxiliary tool to plan cardiac rehabilitation programs, particularly in cases when BET is difficult or impossible to perform. The use of SHAP analysis helped to understand the contribution of each feature to the prediction, increasing the confidence in the model results.

## Introduction

Cardiovascular diseases (CVD) are a serious challenge for healthcare worldwide, being the leading cause of incidence and mortality [1]. According to the World Health Organization, CVD are responsible for 19 million deaths a year, corresponding to nearly 32% of total global mortality [2]. An important element of a complex management of CVD patients is cardiac rehabilitation, providing an integral approach to recovery and secondary prevention. The findings of systematic reviews have indicated the positive effect of cardiac rehabilitation on the survival rate of patients after acute coronary syndrome compared to control groups participating in no rehabilitation programs [3–5]. CVD death rate was found to decrease by 26%, and the frequency rate of readmissions reduced by 18% [6]. The ultimate purpose of cardiac rehabilitation is patient recovery and providing a patient with full reintegration into community without any functional limitations that in total contribute to the improvement of a clinical status, life quality and survival rate prognosis [7].

Different exercise stress tests are used to assess exercise tolerance when appointing trainings in cardiac rehabilitation. A bicycle ergometer (bicycle ergometer test — BET) is used to determine the metabolic equivalent of task (МЕТ) of the taken stress test. However, the method is characterized by relatively high cost and it is time-consuming [8]. Despite the fact BET can be occasionally used as part of cardiac rehabilitation program, a 6-minute walk test (6MWT) is frequently applied for the routine monitoring of patients’ physical capability. 6MWT is an economical, relatively rapid, and safe technique to assess the functional tolerance of exercise in patients with cardiovascular pathologies. Its wide application is due to the possible monitoring of different CVD courses, as well as the efficacy evaluation of therapeutical and rehabilitation procedures [9, 10]. It should be noted that BET and 6MWT are essentially different. BET considers achieving the maximum capacity within 8–12 min by using the protocol with gradually increasing intensity [11], while 6MWT is a test with constant exercise intensity, and traditionally classified as submaximal, making the test safer. In particular, performing a maximum test, such as BET, poses higher risks for patients with severe conditions compared to submaximal exercises (6MWT) [12]. The fact is confirmed by the differences in safety requirements when performing both tests, including the need for qualified staff, a defibrillator, electrocardiographic monitoring, and the first aid supplies. Thus, in clinical practice there is frequently the problem to determine the optimal exercise intensity when carrying out trainings during cardiac rehabilitation, when performing BET is impossible for various reasons.

Over the last years, the interest in artificial intelligence (AI) advances in medical community has grown, that particularly stimulated the introduction of monitoring systems into rehabilitation practice [13]. One of the key areas of AI application is the image processing and categorization that has already been realized in many medical fields, including radiology, oncology, dermatology, and cardiology [14–17]. AI is being increasingly used in robot-assisted surgery, genomics, prognosis of clinical outcomes, and in decision-making processes. The research findings have not infrequently demonstrated AI can achieve the competency proficiency of clinicians and even leave them behind.

Machine learning methods are used for modeling complex interactions between sets of variables [14–17]. Particularly, cardiac rehabilitation efficiency requires the analysis of clinical, psychological, and anthropometric parameters, as well as the characteristics related to risk factors and cardioprotective habits. AI technologies are able to provide the integration of data obtained through different sources, including sensors to record physiological parameters such as blood pressure, heart rate (HR), electrocardiographic data on exertion, oxygen saturation (SpO_2_) in blood, and others. Thus, there is the potential opportunity of BET data prognosis based on different parameters obtained from remote biosensors during a 6MWT.

Despite the growing interest in AI in medicine, there are just several studies in rehabilitation. Near twenty years ago, Zhu et al. [18] in their comparative study involving over 20,000 patients with home care demonstrated that even a simple algorithm of k-nearest neighbors can predict more accurately the rehabilitation potential than standard clinical evaluation reports. In their further research, Zhu et al. [19] stated the support vector machine and random forests to far exceed traditional data analysis techniques applied in rehabilitation.

Lin et al. [20] used machine learning to predict poststroke rehabilitation results. The data analysis of nearly 300 patients using logistic regression, support vector machine, and random forest showed regression algorithms enable to assess Barthel index value with a mean absolute error being around 10, while classifiers provide over 70% accuracy in dividing patients into three categories according to the activity level.

**The aim of the study** was to develop an optimal technique to predict the results of a BET based on the parameters recorded during a 6MWT using machine learning methods. The present study aimed at developing an effective accessory tool for planning cardiac rehabilitation programs.

## Materials and Methods

The present study was prospective, cohort; and analyzed the data of 56 patients with acute myocardial infarction: 45 male (79.4%) and 11 female (20.6%) patients. The study was carried out in Cardiological Dispensary (Russia), where the inpatients had the second stage of cardiac rehabilitation.

All patients underwent a complex examination including: history- and complaint-taking, physical examination, as well as anthropometric assessment (height, body mass, body mass index — BMI). Within 24 h prior to the physical rehabilitation started, all patients had symptom-limited BET and 6MWT. The reason for BET termination was a patient’s refusal of further test performance due to subjective exercise intolerance (expressed fatigue, the pain syndrome in the lower limbs, breathlessness, etc.). The condition was interpreted as achieving the exercise intolerance limit. 6MWT was carried out under US guidance using Accordix — the telemedicine system (Neurosoft, Ivanovo). Accordix is designed for remote monitoring and recording physiological parameters, particularly, ECG at rest and on exertion. The system capabilities include the computer-aided analysis of the recorded data in a real-time mode, including a 6MWT. At an initial stage the following characteristics were assessed: the distance covered by a patient during a 6MWT; the achieved power capacity when performing BET (in MET); the presence of dynamic changes on ECG when performing both tests. Prior to the exercise tests, there were measured HR, arterial blood pressure and SpO_2_. When performing a 6MWT, in addition to the distance covered, we evaluated Borg rating of received exertion (6–20 score scale) and the number of steps taken.

To achieve the target goals, there were developed several machine learning models: random forest regression (RFR), gradient boosting, k-nearest neighbors (kNN) and multiple linear regression (MLR). The machine learning models were developed, and the findings were visually analyzed in programing language Python using Google Colab.

**Figure 1.**
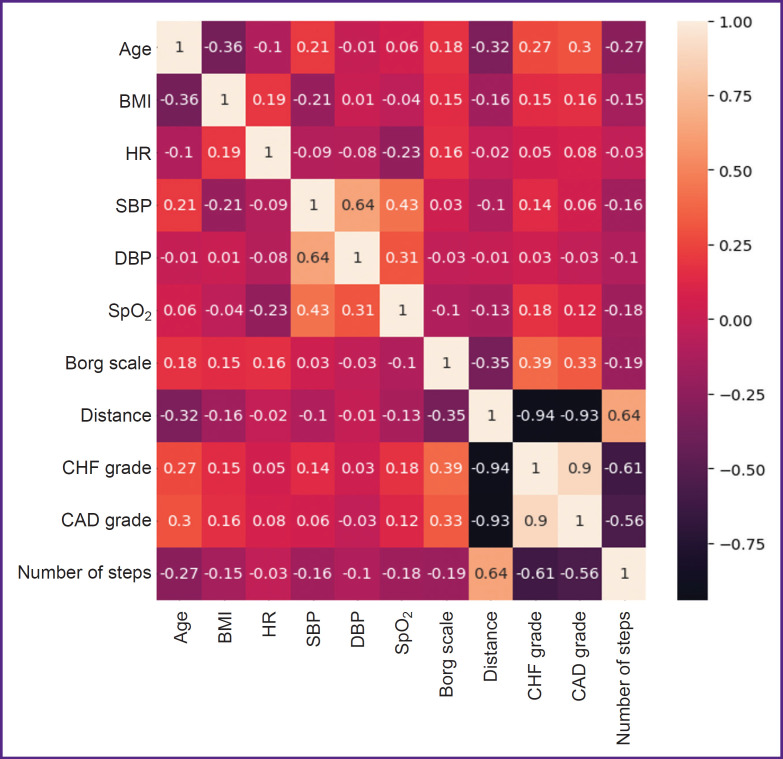
Correlation matrix of the patients’ characteristics under study Age — mean patient age (years); BMI — body mass index; HR — heart rate, prior to a six-minute walk test (6MWT) (bpm); SBP — systolic blood pressure, prior to 6MWT (mm Hg); DBP — diastolic blood pressure, prior to 6MWT (mm Hg); SpO_2_ — oxygen saturation (%); Borg scale — Borg rating of perceived exertion (scores); distance — the distance covered (m); CHF grade — chronic heart failure grade; CAD grade — coronary artery disease grade; number of steps — the number of steps taken

The presence of highly correlating characteristics is known to result in the model performance reduction due to high dispersion and less interpretability [21]. For this reason, the correlation coefficient threshold for the considered features was set as 0.7. In case the threshold was higher, one of the correlating features was excluded. Resulting from plotting the correlation matrix of the initial sampling features, there were found two characteristics, which were excluded from the further analysis: chronic heart failure grade and coronary artery disease grade (Figure 1).

## Results

Two developed models (random forest regression and gradient boosting) are characterized by a sufficiently high determination coefficient. In the models of k-nearest neighbors and multiple linear regression, the coefficient values were less than 0.5 in case of 6MWT value predicted. It means that the share of variational factorial features is the minor part of dispersion compared with the other factors unaccounted in the model and having an effect on the predicted variable change. Regression models developed under such conditions are of low practical importance. The gradient boosting model showed the best results by all characteristics, demonstrating the highest correction values and the lowest error rate (see the Table).

**Table:** Parameters of the predicted models

Models	Six-minute walk test				Metabolic equivalent			
	R^2^	MAE	MSE	RMSE	R^2^	MAE	MSE	RMSE
Random forest regression	0.88	3.43	17.22	4.15	0.90	0.28	0.12	0.35
Gradient boosting	0.99	0.76	0.99	0.99	0.99	0.06	0.01	0.07
k-nearest neighbors	0.46	6.93	75.42	8.68	0.67	0.47	0.39	0.62
Multiple linear regression	0.30	7.81	98.92	9.95	0.52	0.62	0.57	0.75

N o t e: R^2^ — determination coefficient; MAE — mean absolute error; MSE — mean square root error; RMSE — root mean square error.

**Figure 2.**
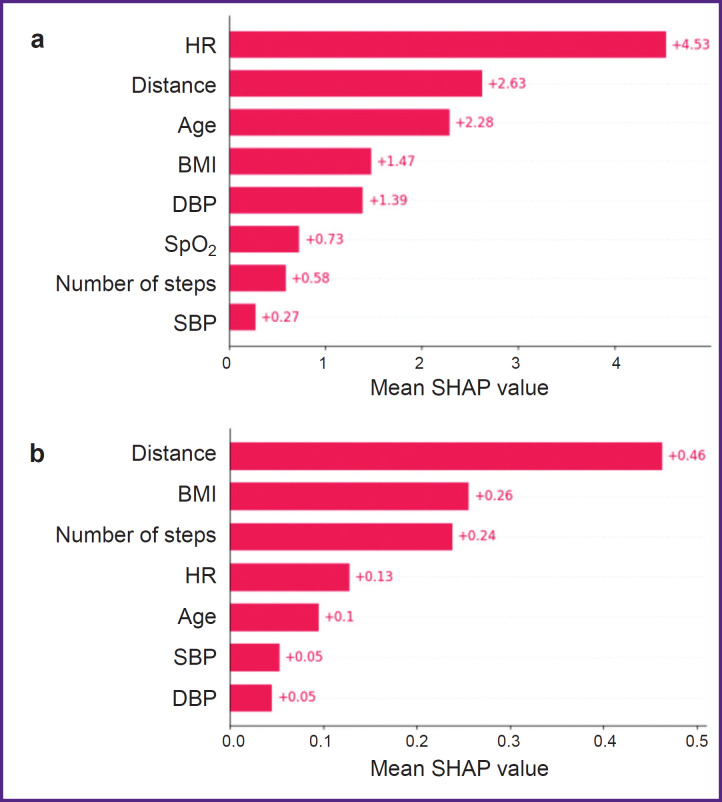
Mean absolute SHAP value in the gradient boosting model for a 6-minute walk test (6MWT) (a) and the metabolic equivalent (b) HR — heart rate, prior to 6MWT (bpm); distance — the distance covered (m); age — mean patient age (years); BMI — body mass index; DBP — diastolic blood pressure, prior to 6MWT (mm Hg); SpO_2_ — oxygen saturation (%); number of steps — the number of steps taken; SBP — systolic blood pressure, prior to 6MWT (mm Hg)

For more detailed analysis of the factors influencing the predicted values in the gradient boosting model, there was developed a column chart of the features sorted out by a mean SHAP value (Figure 2 (a)). It is seen that the absolute significance of some features was rather high. So, to predict 6MWT value, the most significant was HR value with SHAP value — 4.53; the less contribution to prediction was made by the distance covered (SHAP value — 2.63) and the patient age (SHAP value — 2.28). BMI had SHAP value — 1.47. In case of MET prognosis (Figure 2 (b)) the most contribution was made by the distance covered with SHAP value — 0.46; then downwards: BMI (SHAP value — 0.26) and the number of steps taken (SHAP value — 0.24). HR, age, systolic and diastolic blood pressure had the minimum effect on MET value prognosis.

## Discussion

In contrast to the works by De Cannière et al. [22], Desai et al. [23], and Alshurafa et al. [24], who used the support vector machine, random forest, decision trees or neural networks to monitor patients, we used gradient boosting, which is a relatively new approach in this field. De Cannière et al. [22] in the support vector model achieved the mean absolute error — 42.8±36.8 in performance capability prognosis evaluated by 6MWT distance, while the mentioned gradient boosting model demonstrated significantly lower error values for both target metrics (6MWT and MET).

SHAP analysis used to interpret the gradient boosting model findings enabled to determine the most significant variables in BET outcome prognosis. The analysis also revealed the differences in the significance of features to predict 6MWT distance and MET.

The most significant for 6MWT distance prognosis appeared to be HR prior to the test, the patient age, and BMI. The higher BMI is frequently associated with the physical form deterioration, the decreased aerobic capacity and an increased load on the locomotor apparatus that can restrict the covered distance [25]. Initial HR reflects the autonomic nervous tone and the functional condition of the cardiovascular system having an impact on physical efficiency [26]. With age, the body functional reserve decreases, it also has a negative effect on the distance covered [27].

In its turn, the number of steps and BMI appeared to be the most important for MET prognosis. The number of taken steps is directly related to the physical activity level and energy consumption. BMI, similar to 6MWT distance, can restrict the achievement of higher MET levels. It is worth noting that such parameters as HR, systolic and diastolic blood pressure demonstrated less significance. It can be related to their higher variability and the dependence on a number of factors unaccounted in the model.

The clinical significance of the developed gradient boosting model is in its potentiality to optimize cardiac rehabilitation programs. In cases when BET is difficult or impossible to perform (e.g., in severe patients or in case there is no equipment needed), our model can serve as a valuable accessory tool to assess the functional performance and plan individual training programs. It will allow doctors to make more appropriate decisions on physical load intensity, exercise duration and other cardiac rehabilitation aspects that consequently can result in improving the therapy results and patients’ safety. The model can also be used to monitor patients’ progress in cardiac rehabilitation and the early correction of a training program. It can contribute to the improvement of life quality and survival rate prognosis in CVD patients.

The chief limitation of the present study was the relatively small sampling volume that can potentially restrict the generalizability of findings.

## Conclusion

The present prospective cohort study demonstrated the efficiency of using current machine learning methods to predict BET results based on the data obtained by a 6MWT. The application of random forest, gradient boosting, k-nearest neighbors, and multiple linear regression enabled to identify the most informative features, which have an impact on the predicted 6MWT and MET values.

The gradient boosting model was stated to provide the best prognosis quality characteristics with a high determination coefficient (R^2^=about 0.99) and low error values (МAЕ, MSE, RMSE) for both target metrics. It confirms the approach efficiency to predict the results of BET based on 6MWT findings with no need for costly and resource-intensive tests. The method is particularly relevant for severe patients, who can hardly undergo maximum load tests, which can be risk-bearing.
